# SAX-3 (Robo) and UNC-40 (DCC) Regulate a Directional Bias for Axon Guidance in Response to Multiple Extracellular Cues

**DOI:** 10.1371/journal.pone.0110031

**Published:** 2014-10-15

**Authors:** Xia Tang, William G. Wadsworth

**Affiliations:** Department of Pathology, Rutgers Robert Wood Johnson Medical School, Piscataway, New Jersey, United States of America; Trinity College Dublin, Ireland

## Abstract

Axons in *Caenorhabditis elegans* are guided by multiple extracellular cues, including UNC-6 (netrin), EGL-20 (wnt), UNC-52 (perlecan), and SLT-1 (slit). How multiple extracellular cues determine the direction of axon guidance is not well understood. We have proposed that an axon's response to guidance cues can be modeled as a random walk, *i.e.*, a succession of randomly directed movement. Guidance cues dictate the probability of axon outgrowth activity occurring in each direction, which over time creates a directional bias. Here we provide further evidence for this model. We describe the effects that the UNC-40 (DCC) and SAX-3 (Robo) receptors and the UNC-6, EGL-20, UNC-52, and SLT-1 extracellular cues have on the directional bias of the axon outgrowth activity for the HSN and AVM neurons. We find that the directional bias created by the cues depend on UNC-40 or SAX-3. UNC-6 and EGL-20 affect the directional bias for both neurons, whereas UNC-52 and SLT-1 only affect the directional bias for HSN and AVM, respectively. The direction of the bias created by the loss of a cue can vary and the direction depends on the other cues. The random walk model predicts this combinatorial regulation. In a random walk a probability is assigned for each direction of outgrowth, thus creating a probability distribution. The probability distribution for each neuron is determined by the collective effect of all the cues. Since the sum of the probabilities must equal one, each cue affects the probability of outgrowth in multiple directions.

## Introduction

**Figure 1:**
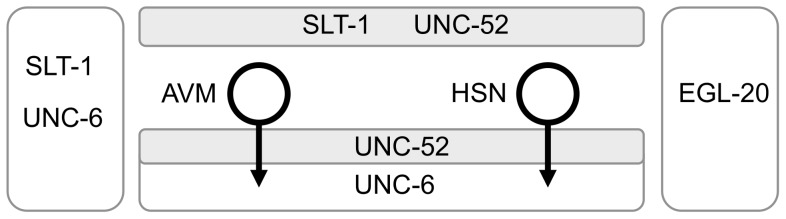
AVM and HSN axons are guided by multiple extracellular cues. (A) Schematic diagram of the position of the AVM and HSN neurons relative to the sources of extracellular molecules that affect axon guidance. The AVM neuron is located on the lateral right side of the body wall, anterior of the vulva. During larval stages, the AVM axon is guided ventrally to the ventral nerve cord, where it turns and migrates anteriorly to the nerve ring. There are two bilaterally symmetric HSN neurons located on the lateral sides of the body wall, posterior of the vulva. HSN axons are also guided during larval stages to the ventral nerve cord, where the axons turn anteriorly and grow to the nerve ring. UNC-6 and SLT-1 are secreted by cells that are ventral and dorsal, respectively, to the cell bodies. Cells in the head also secret the UNC-6 and SLT-1 cues [10]–[12]. EGL-20 is expressed by cells located in the posterior area of the animal near the anus [30], [31]. UNC-52 is strongly associated with the muscle/epidermis extracellular matrix that is ventral and dorsal of the cell bodies [32], [33]. The axons invade this matrix to reach the ventral nerve cord.

In response to multiple extracellular guidance cues an axon is directed towards its target. The extracellular SLT-1 (slit) and UNC-6 (netrin) molecules and the SAX-3 (robo) and UNC-40 (DCC) receptors play a conserved role in the guidance of axons [1]–[7]. The migration of the HSN and AVM axons in *Caenorhabditis elegans* provides a model to study how axons are guided by UNC-6/UNC-40 and SLT-1/SAX-3 signaling. These neurons are at different positions on the lateral body wall, but are exposed to the same extracellular guidance cues, which include UNC-6, SLT-1, UNC-52 (perlecan) and EGL-20 (wnt) (Figure 1). The axons form during larval development and are easily visualized [8], [9]. The axons migrate towards a ventral source of UNC-6 and away from a dorsal source of SLT-1 [10]–[12]. Mutations that affect UNC-6/UNC-40 and SLT-1/SAX-3 signaling prevent the axons from effectively reaching the ventral nerve cord [10]–[17]. It's been shown that for this guidance the SAX-3 and UNC-40 receptors function cell-autonomously within neurons [14], [16].

It is commonly proposed that netrins and slits function as attractants and repellants [18]–[20]. Therefore, HSN and AVM guidance is thought to be the result of attractive UNC-6/UNC-40 and repellent SLT-1/SAX-3 signaling. However, recent experimental evidence suggests that the directional response to UNC-6 is stochastically determined [21], [22]. This was first suggested because of the phenotypes caused by a specific point mutation in *unc-40(ur304)* [22] and by the phenotypes caused by that lack of the cytoskeletal binding protein UNC-53 [21]. In both cases, the asymmetric localization of UNC-40 is induced in the *unc-6* loss-of-function background. However in these mutants, UNC-40 asymmetric localization is directed to a different side of the neuron, which results in the axon protruding from a different side of the neuron in different animals. In the *unc-6* wild-type background, UNC-40 localization and axon protrusion is normal, at the ventral side. The interpretation is that UNC-40 mediates two separate responses. First, UNC-40 mediates a response to the UNC-6 molecule that causes UNC-40 asymmetric localization and, second, UNC-40 mediates a response to the external asymmetric distribution of UNC-6 that orients the asymmetric localization of UNC-40 to a specific side of the neuron. Because UNC-40-mediated axon outgrowth activity can be induced without the UNC-6 extracellular spatial cue, it was hypothesized that the direction of UNC-40 axon outgrowth activity might be stochastically determined [22].

The *unc-40(ur304)* phenotypes suggested that random UNC-40 asymmetric localization within the neuron becomes stabilized at one side of the neuron because of the UNC-6 gradient [22]. Recent live-cell imaging of UNC-40 clustering in the anchor cell of *C. elegans* provides important evidence that this process occurs in cells [23]. However, these experiments do not provide evidence that movement occurs through a stochastic process. In probability theory, a stochastic process is a collection of random variables. A random variable is a variable that can take on a set of possible different values. The possible values of a random variable and their associated probabilities define a probability distribution. Although real-time imaging reveals that UNC-40 localization patterns are dynamic in HSN and the anchor cell [21], [23], these observations can't distinguish between random and oscillatory movement, *i.e.* the localization occurs at a determined site that shifts its position according to some defined, but indiscernible, pattern.

**Figure 2:**
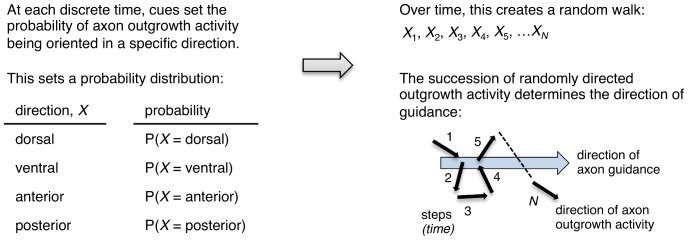
Model for guidance cues creating random walk movement. Guidance cues regulate the probability of axon outgrowth from each side of the neuron. As this occurs in succession over time, movement that can be modeled as a random walk is created. If the probability of axon outgrowth in one direction is greater than the probability of outgrowth in other directions then a bias is introduced that leads to a net drift on average in one direction, which is the direction of axon guidance. Therefore, it is the succession of randomly directed axon outgrowth activity that defines the direction of axon guidance rather than the direction of axon outgrowth at any discrete time.

We have proposed that the movement of axon outgrowth in response to guidance cues can be mathematically described as a stochastic process [21]. This is because we can measure variability at two distinct stages of HSN axon formation [21], [22], [24]. The first stage is when a leading edge forms at one side of the cell body [9]. This event can be visualized by an UNC-40::GFP marker. The second stage is when the single axon develops from the cell body. The direction of outgrowth during these distinct events is a variable. This variable can take a different value (dorsal, ventral, anterior, or posterior) and a probability can be assigned to each of the values, thereby creating a probability distribution (Figure 2).

We further reasoned that the time frame when the direction of outgrowth is measured comprises a series of shorter time intervals. It follows that at each step of this series there is a probability that the direction of axon outgrowth activity will be dorsal, ventral, anterior, or posterior, according to the probability distribution. In mathematics, this is a random walk, *i.e.*, a path made by a succession of randomly directed movement (Figure 2). By this logic, we hypothesize that random walk movement can describe the axon outgrowth activity that occurs in response to guidance cues. Proof for the model comes from evidence that axon development behaves according to a random walk.

In two previous papers [21], [24], we presented experimental evidence that the HSN axon develops in accordance with the properties of random walk movement. One piece of evidence is that the formation of a morphologically mature axon is delayed when the direction of outgrowth activity is variable [21], [24]. For random walks, the mean square displacement (msd) tends to increase only linearly with time, whereas the msd increases quadratically with time for straight-line motion [25]–[27]. Therefore in the mutants, where the direction of axon outgrowth activity randomly fluctuates, an extension can't move as far in the same amount of time.

A second piece of evidence is that the direction of axon outgrowth activity at a discrete time does not determine the direction of guidance. A property of random walk movement is that the direction of movement over time is the consequence of a succession of randomly directed movement. If the direction of axon outgrowth activity stochastically fluctuates in response to guidance cues then the direction of axon outgrowth at a discrete time may not reveal the direction of guidance over time. We observe that in mutants where the axon outgrowth activity fluctuates, the axon outgrowth from the HSN cell body is directed anteriorly or posteriorly although the axon will be guided by UNC-6 to the ventral nerve cord [21], [24].

Here we describe a third piece of evidence; a guidance cue directs axon outgrowth in multiples directions and the direction depends on the other cues. In a random walk there is a probability associated with each direction and the sum of all the probabilities must equal one. The random walk therefore dictates that an individual cue must affect the probability of outgrowth in more than one direction. If a cue increases or decreases the probability of outgrowth in one direction it must affect the probability of outgrowth in another direction(s) as well. The directional affect a cue has depends on the effects other cues have on the probabilities.

By examining loss-of-function mutants in the context of the random walk model, we can make new interpretations of how the UNC-40 and SAX-3 receptors and the UNC-6, EGL-20, UNC-52, and SLT-1 cues affect axon guidance. We find that SAX-3 is required for the induction of the asymmetric localization of UNC-40 in HSN. This induction does not require SLT-1, which is a ligand for SAX-3. We provide evidence that there are different axon outgrowth activities that are regulated by SAX-3 and UNC-40. These can regulate the directional bias in response to specific cues. We also observe cell-specificity; SLT-1 affects the directional bias of AVM but not HSN, whereas UNC-52 affects the directional bias of HSN but not AVM. Together our results suggest a remarkably flexible guidance system based on the properties of random walk movement.

## Results

### SAX-3 and UNC-40 affect the probability of HSN axon outgrowth in each direction

**Figure 3:**
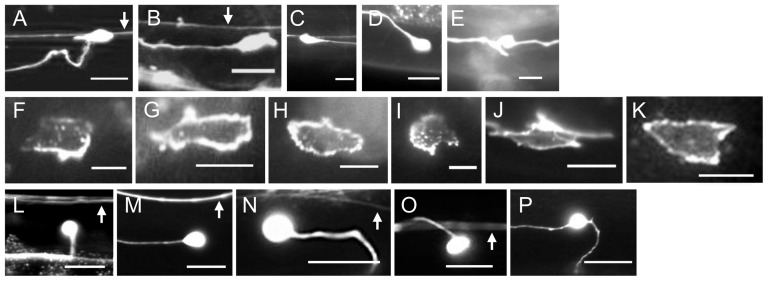
Mutations affect intracellular UNC-40::GFP localization and the direction of axon protrusion. (A–E) Photomicrographs of L4 stage animals showing examples of the protrusion of the axon from the HSN cell body in wild-type and mutant animals. Ventral is down and anterior is to the left. Arrow indicates the PLM axon, if in the focal plane. Scale bar: 10 µm. (A) In wild-type animals, the HSN axon protrudes ventrally from the cell body. After reaching the ventral nerve chord the axon extends anteriorly and defasciculates from the cord to form synapses at the vulva. It then refasciculates with the nerve cord and grows anteriorly to the nerve ring. In mutants, the HSN axon can protrude in the anterior (B), posterior (C), or dorsal (D) directions. In some mutants, the neuron will be bipolar (E). (F–K) Photomicrographs showing examples of UNC-40::GFP localization in the HSN neuron of L2 stage larvae. Ventral is down and anterior is to the left. Scale bar: 5 µm. UNC-40::GFP is ventrally localized in the wild-type animals (F), but in *unc-6(ev400)* mutants (G) and *sax-3* mutants (H) UNC-40::GFP is evenly distributed. In *slt-1* mutants, UNC-40::GFP is ventrally localized (I). In *unc-53;unc-6* double mutants UNC-40::GFP will localize to different sides of the neuron, including the dorsal side (J). However in *unc-53;sax-3;unc-6* triple mutants UNC-40::GFP is evenly distributed (K). (L–P) Photomicrographs of L4 stage animals showing examples of the protrusion of the axon from the AVM cell body in wild-type and mutant animals. Ventral is down and anterior is to the left. Arrow indicates the ALM axon, if in the focal plane. Scale bar: 10 µm. (L) In wild-type animals, the ALM axon protrudes ventrally from the cell body. After reaching the ventral nerve chord the axon extends anteriorly to the nerve ring. In mutants, the ALM axon can protrude in the anterior (M), posterior (N), or dorsal (O) direction. In some mutants, the neuron will be bipolar (P).

We analyzed how SAX-3 and UNC-40 affect the probability of axon outgrowth in each direction from the HSN cell body. It is common to study genes that are suspected of affecting HSN guidance by assaying whether or not the axon reaches the ventral midline in a mutant. This model assumes that the neurons have a specific directional response to a guidance cue. However, if the directional response to the guidance cue is stochastic and there is random walk movement, then the direction of axon guidance is determined by the succession of randomly directed outgrowth. Therefore, to understand how a gene affects the direction of axon guidance it is important to understand how it affects random walk movement. We therefore measured how a mutation affects the probability of outgrowth in four directions from the HSN cell body (Figure 3 A–E). This information can then be used to simulate a simple random walk. The results estimate how the mutation would alter the direction of guidance if the probabilities were kept constant over time. That is, the results graphically show the directional bias that the cues created as the axon protruded from the cell body.

In this study we used two alleles of *sax-3*. The *sax-3(ky123)* allele results in a deletion of the signal sequence and first exon of the gene, whereas *sax-3(ky200)* contains a missense mutation at a conserved proline residue (P37S) in the first immunoglobin-like domain (Ig1) [13]. The *sax-3(ky200)* mutation is temperature sensitive and evidence suggests that the SAX-3 protein in the mutant is misfolded and mislocalized at the restrictive temperature (25°C) [28]. Because *unc-6* and *sax-3* are on the same chromosome and *sax-3(ky123);unc-6(ev400)* doubles do not grow well, it is easier to use the temperature sensitive *sax-3* allele to make doubles with *unc-6(ev400)*.

We find that SAX-3 regulates the probability of axon outgrowth in each direction differently than UNC-40. In comparison to *unc-40(e1430)* and *unc-6(ev400)* loss-of-function mutants, in the *sax-3* mutant the probability of ventral protrusion is greater, whereas the probability of anterior protrusion is less (Table 1). The probability of outgrowth is different in the double mutant, *unc-40(e1430); sax-3(ky200)* than in either single mutant.

**Table 1:** Axon Protrusion from the HSN Cell Body.

	direction of axon protrusion						
HSN phenotype	dorsal	ventral	anterior	posterior	multi	n	ref
*wild-type*	0	96±1	3±1	0	1±1	221	
*unc-6(ev400)*	2±2	3±2	81±2	8±2	6±1	218	[21]
*unc-40(e1430)*	2±1	6±2	67±2	19±1	6±1	183	[21]
*unc-40(ur304)*	0	98±1	2±1	0	0	244	
*sax-3(ky123)*	2±1	31±1	21±1	37±2	9±2	232	
*sax-3(ky200)* *	2±1	32±1	19±2	42±3	5±2	198	
*slt-1(eh15)*	0	95±1	2±1	2±2	1±1	140	
*unc-6(ev400);slt-1(eh15)*	6±2	2±1	65±3	16±2	11±2	176	
*unc-40(e1430);slt-1(eh15)*	12±2	2±1	63±4	17±1	7±4	192	
*unc-52(e444)*	0	60±2	28±2	8±2	4±1	96	[24]
*unc-52(e444); unc-6(ev400)*	13±2	2±1	34±3	24±3	27±3	149	[24]
*unc-40(e1430); unc-52(e444)*	17±2	4±1	30±2	25±2	24±2	153	
*egl-20(n585)*	0	64±2	21±2	7±1	8±1	304	[21]
*egl-20(n585); unc-6(ev400)*	18±2	0	43±2	15±2	24±2	205	[21]
*unc-40(e1430); egl-20(n585)*	6±2	17±2	45±5	15±2	16±2	173	[21]
*unc-53(n152)*	0	67±3	22±2	5±1	6±1	238	[21]
*sax-3(ky200)* * *; unc-6(ev400)*	8±1	8±2	49±3	20±5	14±2	211	
*unc-53(n152);sax-3(ky123)*	1±1	47±3	24±2	23±5	6±3	207	
*unc-52(e444);sax-3(ky123)*	1±1	35±2	21±1	40±1	3±2	144	
*unc-40(ur304);sax-3(ky123)*	2±1	37±2	20±3	37±3	4±2	178	
*unc-40(e1430);sax-3(ky200)* *	14±3	2±1	40±2	35±3	9±4	191	
*egl-20(n585);sax-3(ky123)*	1±1	12±2	39±2	39±1	8±3	177	
*unc-53(n152);unc-6(ev400)*	20±2	1±1	27±2	22±2	30±3	174	
*unc-53(n152);sax-3(ky200)* * *;unc-6(ev400)*	11±2	2±1	33±4	30±3	25±5	189	
*unc-40(ur304);unc-6(ev400)*	11±1	4±1	60±2	15±1	11±1	178	
*unc-40(ur304);sax-3(ky200)* * *;unc-6(ev400)*	7±3	4±2	46±4	25±3	17±2	193	

Numbers represent percentage value ± SEM.

*Animals grown at the *sax-3(ky200)* restrictive temperature (25°C).

We also examined *sax-3* double mutants with *unc-52(e444)* and *egl-20(n585)* (Table 1). Whereas there is a strong bias for ventrally directed protrusion in the *unc-52(e444)* and *egl-20(n585)* mutants, the probability of ventral outgrowth decreases in the double mutants. Although SLT-1 is a ligand for SAX-3, we find that in *slt-1(eh15)* mutants the probability of axon outgrowth in each direction is nearly that of wild-type (Table 1). This result suggests that SLT-1 has little influence on the initial guidance of the HSN axon. Overall, our results show that SAX-3 affects the probability of axon outgrowth in each direction differently than UNC-40, and that the effect doesn't require SLT-1 activity.

### Guidance cues have different effects on the probability of axon outgrowth in each direction for HSN and AVM

Previously we reported that mutations that disrupt contact with a ventral extracellular matrix affect UNC-6 axon guidance for the HSN axon, but not for the AVM axon [24]. This suggests that the effects of mutations that perturb UNC-6 guidance can be neuron-specific. Therefore, we decided to determine how mutations affect the direction of outgrowth from AVM (Figure 3 L–P).

We find that in the *sax-3* mutants the probability of AVM axon outgrowth in each direction is similar to that observed in *unc-6(-)* and *unc-40(-)* mutants (Table 2). Specifically, there is a probability for ventral protrusion and a probability for anterior protrusion. As with HSN, in *sax-3(ky200); unc-6(ev400)* mutants the probability of ventral outgrowth decreases, whereas the probability of outgrowth in other directions increases.

**Table 2:** Axon Protrusion from the AVM Cell Body.

	direction of axon protrusion						
AVM phenotype	dorsal	ventral	anterior	posterior	multi	n	ref
*wild-type*	0	100	0	0	0	143	
*unc-6(ev400)*	0	62±2	38±1	0	0	226	[21]
*unc-40(e1430)*	0	76±1	28±3	0	0	257	
*sax-3(ky200)* *	0	58±1	35±2	7±1	0	146	
*sax-3(ky123)*	0	59±2	41±2	0	0	162	
*slt-1(eh15)*	0	58±2	42±2	0	0	168	
*sax-3(ky200)* * *; unc-6(ev400)*	0	34±2	59±3	6±2	1±1	196	
*unc-40(e1430);sax-3(ky200)* *	0	40±3	57±4	1±1	1±1	211	
*unc-53(n152);sax-3(ky123)*	0	47±2	49±3	1±1	2±1	176	
*unc-6(ev400);slt-1(eh15)*	9±2	8±1	83±3	0	0	254	
*unc-40(e1430);slt-1(eh15)*	17±4	12±2	67±3	0	3±2	219	
*egl-20(n585)*	0	89±2	9±2	0	1±1	168	
*egl-20(n585); unc-6(ev400)*	0	49±1	34±2	13±2	4±1	162	
*egl-20(n585);sax-3(ky123)*	0	48±4	50±3	2±1	0	148	
*unc-53(n152)*	0	100	0	0	0	207	[21]
*unc-40(e1430);unc-6(ev400)*	0	70±2	30±2	0	0	174	[21]
*unc-40(ur304)*	0	100	0	0	0	185	
*unc-53(n152);unc-6(ev400)*	14±1	27±2	43±2	12±3	3±2	139	
*unc-40(ur304);unc-6(ev400)*	9±2	60±3	22±2	9±1	1±1	173	
*unc-53(n152);sax-3(ky200)* * *;unc-6(ev400)*	1±1	19±3	65±4	9±2	5±2	222	
*unc-40(ur304);sax-3(ky200)* * *;unc-6(ev400)*	1±1	38±6	52±5	4±2	4±1	225	

Numbers represent percentage value ± SEM.

*Animals grown at the *sax-3(ky200)* restrictive temperature (25°C).

A significant difference between HSN and AVM is the effect that SLT-1 has on axon guidance. Similar to *sax-3* mutants, we find that in *slt-1* mutants the probability of anteriorly directed AVM axon outgrowth increases relative to wild-type animals whereas the probability of ventral outgrowth decreases. There is a notable difference between *sax-3(ky200);unc-6(ev400)* and *slt-1(eh15);unc-6(ev400)*; in the *slt-1(eh15);unc-6(ev400)* mutants there is a higher probability for dorsal and anterior outgrowth and a lower probability for ventral outgrowth. Similarly, *unc-40(e1430);sax-3(ky200)* and *unc-40(e1430);slt-1(eh15)* are different. These results suggest that SLT-1 has an effect on the direction of axon outgrowth that is not mediated by SAX-3.

### SAX-3 and UNC-40 signaling regulate the directional bias

To visualize the effects that a mutation has on a directional bias for axon outgrowth, we simulated a random walk of 250 steps based on the probability of dorsal, ventral, anterior, and posterior outgrowth given in Tables 1 and 2. For each mutant, 10 simulations are plotted. The result gives a prediction of the effect a mutation has on the direction of axon outgrowth during the initial protrusion from the cell body. As well, the distance of the lines from the origin gives a prediction about the nature of the fluctuations caused by a mutation.

**Figure 4:**
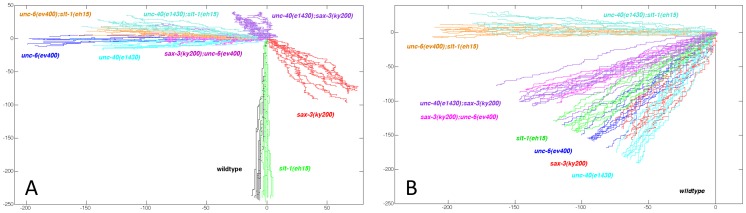
The directional bias during the initial protrusion is controlled by UNC-6 and SLT-1 through UNC-40 and SAX-3 signaling. 10 simulated random walks of 250 steps were plotted from an origin (0, 0). The walks were generated using the probabilities of outgrowth in the dorsal, ventral, anterior, and posterior direction, as listed for each mutant in Table 1. (A) Plots generated to compare the directional bias produced by the different mutations for HSN axon guidance. (B) Plots generated to compare the directional bias produced by the different mutations for AVM axon guidance. Whereas SLT-1 affects the ventral bias for AVM, it has little effect on the directional bias for HSN.

The graphs illustrate that the UNC-40 and SAX-3 receptors mediate a directional bias for AVM and HSN. Whereas in wild-type animals there is a strong ventral directional bias, the directional bias for AVM and HSN in *unc-40(e1430), sax-3(ky200)*, and *unc-40(e1430);sax-3(ky200)* mutants is different (Figures 4A and 4B). In the *sax-3* mutants there is a weaker ventral bias for axon guidance. This ventral bias is further reduced or is eliminated in *unc-40(e1430);sax-3(ky200)* mutants. Further, for HSN there is no ventral bias in *sax-3(ky200);unc-6(ev400)* or *unc-40(e1430)* mutants. Together these results suggest that in the *sax-3(ky200)* mutants UNC-40 mediates a response to UNC-6 that causes a ventral directional bias.

### Loss of UNC-52 and EGL-20 create a directional bias that requires SAX-3 and UNC-40 signaling

**Figure 5:**
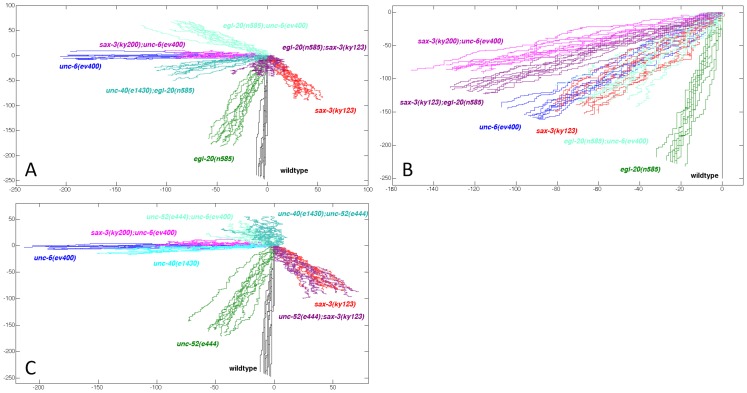
The directional bias during the initial protrusion is controlled by the extracellular cues, UNC-6, EGL-20, and UNC-52. 10 simulated random walks of 250 steps were plotted from an origin (0, 0). The walks were generated using the probabilities of outgrowth in the dorsal, ventral, anterior, and posterior direction, as listed for each mutant in Table 1. (A and B) Plots generated to compare the directional bias caused by *egl-20* in different genetic backgrounds for the HSN (A) and AVM (B) axon. (C) Plots generated to compare the directional bias for HSN axon guidance caused by *unc-52* in different genetic backgrounds. UNC-52 does not affect the directional bias for AVM axon guidance [24].

We previously found that UNC-52 (perlecan) and EGL-20 (wnt) also control the probability of UNC-40 localizing to the ventral side of the HSN neuron. From our analyses of the directional bias, we observe that the directional bias in the double *egl-20(n585);sax-3(ky123)* mutant and the *unc-40(e1430);egl-20(n585)* are not the same as that caused by the loss of EGL-20 alone (Figure 5A and 5B). These phenotypes indicate that the directional bias caused by the loss of EGL-20 requires UNC-40 and SAX-3.

We observe that there is a ventral direction bias in both *sax-3* and *unc-52* mutants (Figure 5C). However, the pattern for *sax-3* mutants is shifted right (posterior), whereas the pattern for *unc-52* mutants is shifted left (anterior). The directional bias of *unc-52* mutants is different from that in the *unc-52(e444);sax-3(ky123)* mutant, suggesting that the directional bias created by the loss of UNC-52 requires SAX-3 activity. We also find that the directional bias in the double *unc-52(e444);sax-3(ky123)* mutant is similar to the bias of *sax-3* mutants, suggesting that UNC-52 affects the directional bias through something that is missing because of the *sax-3(ky123)* mutation. We also note that the loss of both UNC-52 and UNC-40 creates a directional bias that is different form the directional bias created by the loss of only UNC-52 (Figure 5C). This suggests that the directional bias created by the loss of UNC-52 also requires UNC-40 activity.

### The loss of one cue creates a directional bias that requires the other cues

We observe that the directional bias created in the *unc-6(ev400)*, in the *egl-20(n585)*, and in the *egl-20(n585):unc-6(ev400)* mutant are each different (Figure 5A and 5B). Therefore the directional bias caused by the loss of UNC-6 requires EGL-20 and the directional bias caused by the loss of EGL-20 requires UNC-6. Similarly, the directional bias for HSN in the *unc-6(ev400)*, in the *unc-52(e444)*, and in the *unc-52(e444);unc-6(ev400)* mutant are each different (Figure 4C), indicating that the direction bias caused by the loss of UNC-6 requires UNC-52 and the directional bias caused by the loss of UNC-52 requires UNC-6. Finally, for AVM the directional bias in the *unc-6(ev400)*, in the *slt-1(eh15)*, and in the *unc-6(ev400); slt-1(eh15)* mutant are each different (Figure 4B), indicating that the direction bias caused by the loss of UNC-6 requires SLT-1 and the directional bias caused by the loss of SLT-1 requires UNC-6.

These results also show that a cue can be required for guidance in different directions, depending on the presence of another cue or receptor. For HSN axon guidance, UNC-6 is required for a ventral bias in wild-type animals but is required for a posterior bias in *sax-3* mutants (Figure 4A). UNC-52 promotes a ventral bias in wild-type animals and an anterior bias in *unc-6* mutants (Figure 5C). EGL-20 can promote a ventral bias in wild-type animals, a posterior bias in *sax-3* mutants, and an anterior bias in *unc-6* mutants (Figure 5A).

### UNC-40 asymmetric localization is not induced in *sax-3* mutants

During HSN axon formation, extracellular guidance cues polarize axon outgrowth activity within the neuron [9], [29]. In response to UNC-6 the UNC-40 receptor becomes localized to the ventral site during the formation of the leading edge [9]. This process can be visualized using a GFP-tagged UNC-40 (Figure 3F–K) [9].

**Figure 6:**
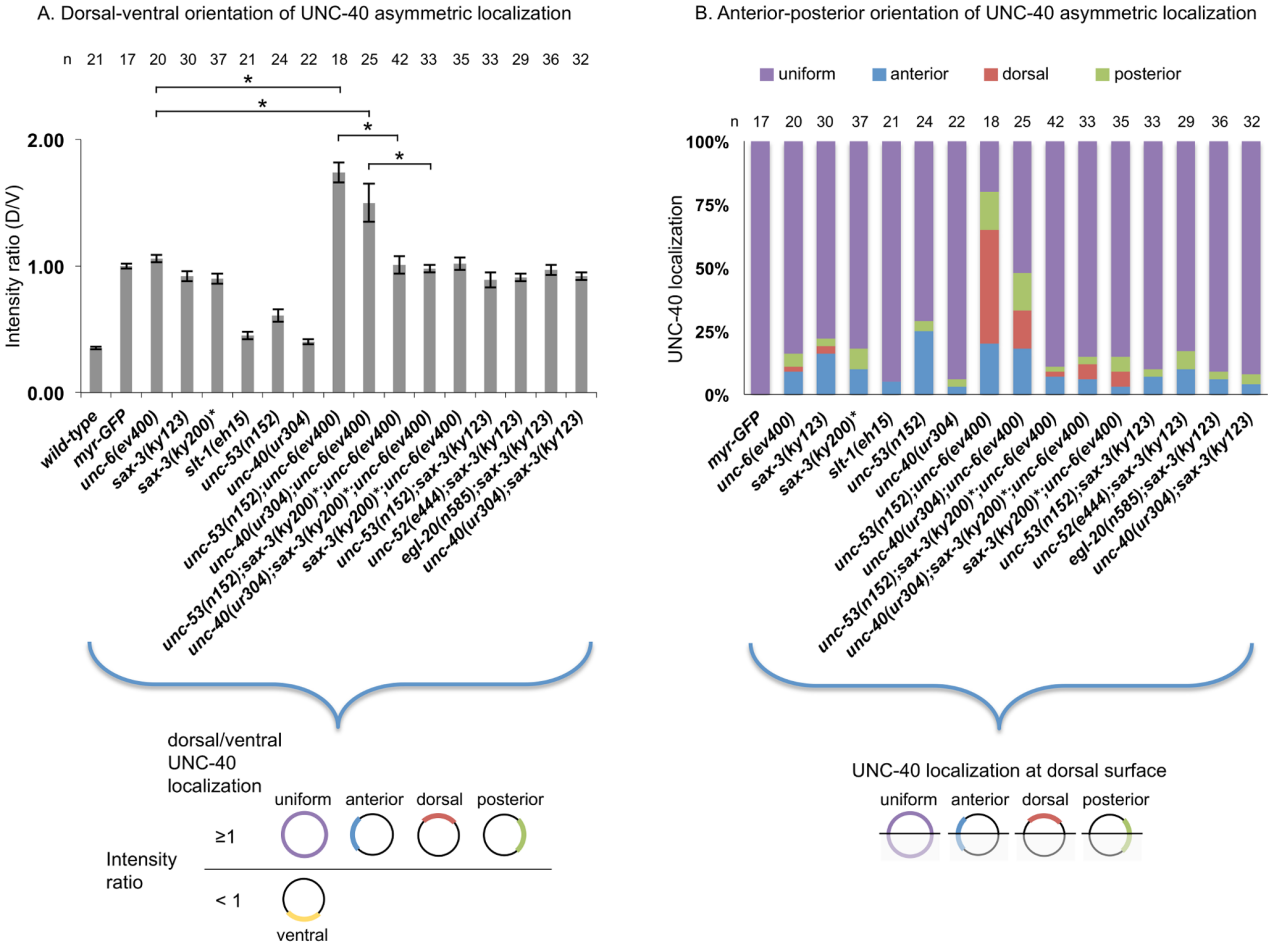
Mutations affect the probability of intracellular UNC-40 localization to different sides of the HSN neuron. (A) Graph indicating the dorsal-ventral localization of UNC-40::GFP in HSN. The graph shows the average ratio of dorsal-to-ventral intensity from linescan intensity plots of the UNC-40::GFP signal around the periphery of the HSN cell. UNC-40::GFP is ventrally localized in wild-type, but the ratio is different in *unc-6(-)* and the mutants. (*) statistic difference (P<0.05, one-tailed Student's *t-*test). Error bars represent standard error of mean. Below is a graphic representation of the possible UNC-40 localization patterns when the intensity ratio is ≥1 or is <1. (B) Graph indicating the anterior-posterior localization of UNC-40::GFP. To determine orientation, line-scan intensity plots of the UNC-40::GFP signal across the dorsal periphery of the HSN cell were taken, the dorsal surface was geometrically divided into three equal segments, and the total intensity of each was recorded. The percent intensity was calculated for each segment and ANOVA was used to determine if there is a significant difference between the three segments (see Material and Methods). In the mutants there is a bias for anterior or posterior localization, whereas there is a uniform distribution in *unc-6(-)* mutants and in double mutants with *unc-6(-)* or *sax-3(-)*. (*) Animals grown at the *sax-3(ky200)* restrictive temperature (25°C). Below is a graphic representation of the possible UNC-40 localization patterns.

UNC-40::GFP remains uniformly dispersed around the periphery of HSN in *unc-6* loss-of-function mutants [9], [22] and we now find that UNC-40::GFP also remains uniformly dispersed around the periphery of HSN in *sax-3* loss-of-function mutants (Figure 3H, G). In these experiments we first assay for the distribution of UNC-40::GFP along the dorsal-ventral axis (Figure 6A). We then assay for bias along the anterior-posterior axis by determining the UNC-40::GFP distribution along the dorsal side of the neuron (Figure 6B).

To further examine the affects of SAX-3 on UNC-40 signaling we examined whether the loss of *sax-3* function can suppress UNC-40 asymmetric localization when the UNC-40 signal is constitutively active. Our previous results indicate that UNC-40 conformational changes can induce the asymmetric localization of UNC-40-mediated axon outgrowth in the absence of UNC-6 [22]. The *unc-40(ur304)* mutation encodes an UNC-40 variant, UNC-40 (A1056V), which can induce the asymmetrical localization of UNC-40 in *unc-6(-)* mutants. In the *unc-40(ur304);unc-6(ev400)* mutants, there is a probability that UNC-40 will localize to any one side of the neuron (Figure 6). The direction of HSN axon outgrowth varies (Table 1). It is proposed that the conformation of UNC-40 (A1056V) partly mimics the confirmation of UNC-40 when UNC-6-ligated. This change allows UNC-40 asymmetric localization to be induced even in the absence of UNC-6. Because the extracellular UNC-6 gradient is missing in *unc-40(ur304);unc-6(ev400)* mutants there is no strong bias for ventral localization and UNC-40 is stochastically directed to any one side of the neuron. We find that in the triple mutant, *unc-40(ur304);sax-3(ky200);unc-6(ev400)* grown at 25°C, UNC-40::GFP remains uniformly dispersed around the periphery of HSN (Figure 6). This result suggests SAX-3 is required for the UNC-40 (A1056V) signal that induces UNC-40 asymmetric localization.

We previously found that the asymmetric localization of UNC-40 in *unc-6(-)* mutants can also be triggered by mutations in *unc-53*, which encodes a cytoskeletal binding protein [21]. UNC-53 appears to function downstream of UNC-40 to mediate the induction signal [21]. We find that in the triple mutant, *unc-53(n152);sax-3(ky200);unc-6(ev400)* grown at 25°C, UNC-40::GFP remains uniformly dispersed around the periphery of HSN (Figure 6). This result suggests SAX-3 is required for the UNC-40 signal that induces UNC-40 asymmetric localization.

**Figure 7:**
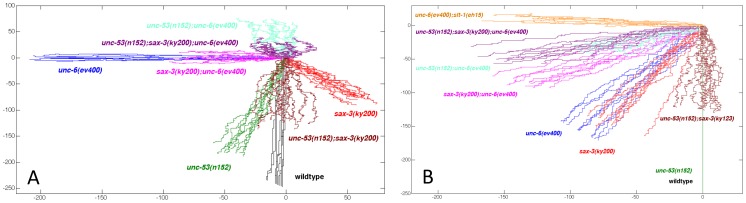
A different directional bias is caused by the loss of UNC-53, by the loss of SAX-3, or by the loss of both. 10 simulated random walks of 250 steps were plotted from an origin (0, 0). The walks were generated using the probabilities of outgrowth in the dorsal, ventral, anterior, and posterior direction, as listed for each mutant in Table 1. (A and B) Plots generated to compare the directional bias caused by *unc-53* and *sax-3* in different genetic backgrounds for the HSN (A) and AVM (B) axon. The direction bias in the *unc-53* and *unc-53;sax-3* double mutants are different and each depends on *unc-6* and *unc-40* activity.

We also examined how the *unc-53* mutation affects the directional bias since UNC-40 asymmetrically localizes in the *unc-53(n152)* and the *unc-53(n152);unc-6(ev400)* mutant but doesn't asymmetrically localized in either the *unc-53(n152);sax-3(ky123)* or the *unc-53(n152);sax-3(ky200);unc-6(ev400)* mutant. We find that the directional bias created in each mutant is different (Figure 7A and 7B). This suggests that the effect that UNC-40 asymmetric localization has on the directional bias depends on the other cues.

We examined whether mutations that alter the probabilities of UNC-40-mediated axon outgrowth occurring in each direction have any affect on UNC-40 asymmetric localization in the *sax-3(-)* mutant. If the loss of SAX-3 inhibits the induction of UNC-40 asymmetric localization in HSN, then UNC-40 localization in the double mutants should be similar to that in the *sax-3* mutant. In comparison to wild-type animals, in *egl-20(n585)* and *unc-52(e444)* mutants the probability of UNC-40 asymmetrically localizing in the anterior and posterior direction increases, whereas the probability of UNC-40 asymmetrically localizing in the ventral direction decreases. We find that in the double mutants, *egl-20(n585);sax-3(ky123)* and *unc-52(e444); sax-3(ky123)*, UNC-40::GFP remains uniformly dispersed around the periphery of HSN (Figure 6). These results are consistent with the idea that SAX-3 is required to induce the UNC-40 asymmetric localization and that this induction is a necessity for the process that determines the probability of where UNC-40 localizes.

### UNC-40 asymmetric localization is induced in *slt-1* mutants

Since SLT-1 is a ligand for SAX-3 we tested whether SLT-1 is also required to induce the asymmetric localization of UNC-40 in HSN. We examined *slt-1(eh15)* mutants and found that the intracellular localization pattern is similar to wild type; UNC-40 does asymmetrically localize to the ventral side of HSN (Figure 6). The *slt-1(eh15)* allele is a deletion that truncates SLT-1 near the N terminus and is a strong loss of function mutation that has been used to inactivate SLT-1 activity [10], [14]. This result indicates that the ability of SAX-3 to induce the UNC-40 signaling is independent of SLT-1.

### Loss of SAX-3 delays HSN axon formation

By the early L2 larval stage, HSN is polarized ventrally with neurites primarily restricted to the ventral side where a leading edge forms [9]. Around the L3–L4 transition a single ventral axon becomes evident. However in *unc-6* and *unc-40* mutants axon development is delayed and neurite extension is not confined to the ventral side during the L3 stage. A predominate axon, which protrudes anteriorly, eventually forms however not until the L4 stage [9]. We hypothesize that the delay is because the direction of axon outgrowth activity randomly fluctuates [21]. Movement in which the direction stochastically fluctuates has distinct properties. A characteristic of a random walk is that the mean square displacement (msd) will increase only linearly with time, whereas the msd increases quadratically with time for straight-line motion. Based on this property, it would be expected that on average it would take longer for an axon to develop in a mutant where the outgrowth activity fluctuates than in a wild-type animal since, given the same amount of outgrowth activity, an axon could not extend as far in the same amount of time.

**Figure 8:**
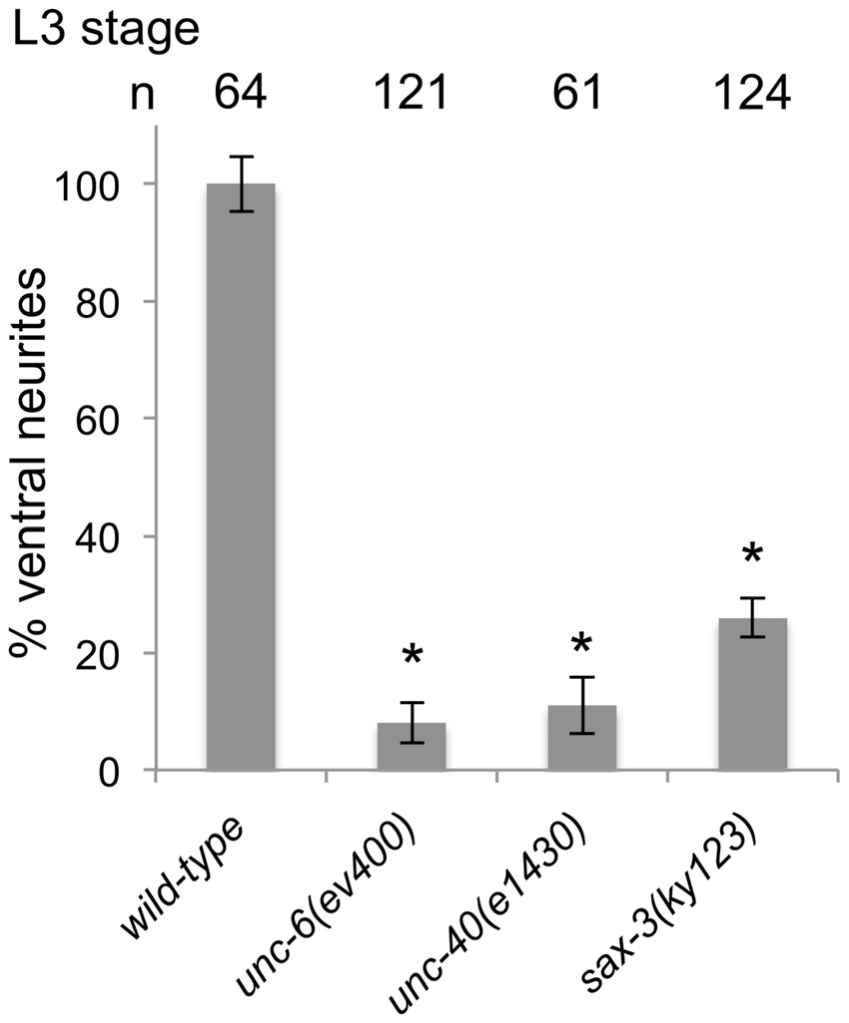
Loss of SAX-3 delays the development of predominantly ventral neurites. (A) The percentage of HSN neurons with predominantly ventral neurites in mid-L3. Whereas in wild-type animals there are predominately ventral neurites by this stage, in *unc-6(ev400)*, *unc-40(e143)*, and *sax-3(ky123)* mutants there is a delay. In all strains an axon will form in the L4 stage. Error bars indicated the standard error mean; n values are indicated above each column. Significant differences (two-tailed *t*-test), *P<0.001.

Axon outgrowth activity can fluctuate in the *sax-3(ky123)* mutant (Table 1) and we observe, similar to the *unc-6(ev400)* and *unc-40(e1430)* mutants, that the neurons don't clearly show ventrally oriented neurites until around the L3–L4 transition (Figure 8).

## Discussion

### The effects that the loss of cues have on the direction of guidance is consistent with random walk movement

Previously we presented evidence that UNC-6 induces axon outgrowth activity that is stochastically directed towards each side of the neuron and that external cues, such as the UNC-6 gradient, regulate the probability that axon outgrowth activity will be directed to each side of the neuron [21], [22]. We proposed that the neuron's response to extracellular cues could be modeled as random walk movement. Guidance cues dictate the probability of axon outgrowth activity occurring in each direction, which over time creates a directional bias. We previously described several behaviors of axon development that are consistent with the properties of random walk movement. Here we describe the effects that the UNC-40 (DCC) and SAX-3 (Robo) receptors and the UNC-6, EGL-20, UNC-52, and SLT-1 extracellular cues have on creating a directional bias. We find that each cue is required for guidance in multiple directions. The direction of guidance depends on the combination of cues.

### Analyzing SAX-3 phenotypes in the context of random walk movement provides new interpretations for how SAX-3 and UNC-40 can affect axon guidance

We find that SAX-3 is required for the process that induces UNC-40 asymmetric localization. In loss-of-function *sax-3* mutants UNC-40 remains uniformly dispersed around the periphery of HSN, as it does in *unc-6(-)* mutants (Figure 6). Further, the *sax-3* mutations suppress UNC-40 asymmetric localization caused by *unc-40(ur304);unc-6(ev400)* or *unc-53(n152);unc-6(ev400)*. Because of these observations, we deduce that UNC-40-based axon outgrowth does not play a significant role in determining the direction of axon guidance in *sax-3* mutants. This further suggests that in *sax-3* mutants non-UNC-40-based axon outgrowth activity directs axon outgrowth. We further observe that there is a loss of ventral directional bias in *unc-53(n152);sax-3(ky200);unc-6(ev400)* mutants (Figure 5C), suggesting that UNC-6 does guide the axons in the *unc-53(n152);sax-3(ky123)* mutant. This result is consistent with different UNC-40 signals controlling induction and orientation. In the *unc-53(n152);sax-3(ky123)* mutant the loss of SAX-3 function interferes with the induction signal and prevents UNC-40 asymmetric localization, but it does not inhibit the UNC-40 signaling that orients axon outgrowth activity in response to the UNC-6 gradient. These observations suggest that SAX-3 could play a role in balancing UNC-40 signaling that induces UNC-40 asymmetric localization with UNC-40 signaling that orients axon outgrowth activity. Possibly, the uniform distribution of UNC-40 at the surface of the cell is required for the orientation signal.

We further observe that the directional bias in *sax-3* mutants required EGL-20 and UNC-6 (Figure 5A and 5B), however it does not require UNC-52 (Figure 5C). We also note that the directional bias in an *unc-52* mutant does require UNC-40. We hypothesize that EGL-20 and UNC-6 direct non-UNC-40-based axon outgrowth activity in the *sax-3* mutant, however UNC-52 can only directs UNC-40-based axon outgrowth activity and so it has no affect on the directional bias. From these observations we suggest that there are different axon outgrowth activities and that the extracellular cues can affect each differently.

### Analyzing SLT-1 phenotypes in the context of random walk movement provides new interpretations for how SLT-1 and UNC-40 can affect axon guidance

We observe a ventral directional bias for AVM in *sax-3(ky200);unc-6(ev400)* mutants but not in *unc-6(ev400);slt-1(eh15)* or *unc-40(e1430);slt-1(eh15)* mutants (Figure 4B). These results suggests that the AVM ventral directional bias observed in *sax-3(ky200);unc-6(ev400)* is caused by SLT-1 signaling that is mediated by UNC-40. The ability of the UNC-40 receptor to mediate a response to SLT-1 was also hypothesized based on the observation that some *slt-1* gain-of-function phenotypes can be suppressed by *unc-40* loss-of-function mutations [14]. Since SLT-1 does not affect UNC-40 asymmetric localization in HSN (Figure 6) and since UNC-40-based axon outgrowth does not appears to play a significant role in determining the direction of axon guidance in *sax-3* mutants, we infer that the directional bias for the AVM axon in *sax-3(ky200);unc-6(ev400)* mutants is because UNC-40 mediates a response to SLT-1 that orients the non-UNC-40 axon outgrowth activity.

STL-1 is a ligand for SAX-3-mediated guidance [10]. Genetic evidence for STL-1/SAX-3 signaling stems from the observation that the ability of the AVM axon to extend to the ventral nerve chord in the *sax-3(ky123); slt-1(eh15)* double mutant is not more defective than the *slt-1(eh15)* single mutant [10]. We observe that the AVM ventral directional bias in *unc-40* mutants, which must be caused by non-UNC-40-based axon outgrowth activity, is reduced in *unc-40(e1430);sax-3(ky200)* mutants (Figure 4B). This suggests the ventral directional bias in *unc-40* mutants requires SAX-3. We also note that there is a ventral directional bias in *unc-6(ev400)* mutants but not in *slt-1(eh15);unc-6(ev400)* mutants, suggesting that SLT-1 causes the ventral directional bias in *unc-6* mutants. STL-1 in the *unc-6* mutants could cause the directional bias either through the UNC-40 or SAX-3 receptor, however a similar ventral directional bias can be derived for *unc-40(e1430);unc-6(ev400)* and *unc-6(ev400)* mutants (Table 2). Together these results provide further genetic evidence that SLT-1 also acts through SAX-3 signaling to determine the direction of guidance.

## Materials and Methods

### Strains

Strains were handled at 20°C by using standard methods (Brenner, 1974) unless stated otherwise. A Bristol strain N2 was used as wild type. The following strains were used: **LGI**, *unc-40(e1430), unc-40(ur304), zdIs5[mec-4::GFP]*; **LGII**, *unc-52(e444), unc-53(n152)*; **LGIV**, *egl-20(n585)*, *kyIs262[unc-86::myr-GFP;odr-1::dsRed]*; **LGX**, *unc-6(ev400)*, *sax-3(ky123), sax-3(ky200), slt-1(eh15)*.

Transgenes maintained as extrachromosomal arrays included: *kyEx1212 [unc-86::unc-40-GFP;odr-1::dsRed]*.

### Analysis of the timing of HSN axon outgrowth

HSN neurons were visualized using the transgenic strain *kyIs262[unc-86::myr-GFP]*. Synchronized worms were obtained by allowing eggs to hatch overnight in M9 buffer without food. L3 staged nematodes larvae were mounted on a 5% agarose pad with 10 mM levamisole buffer. Larval staging was determined by the gonad cell number and gonad size under differential interference contrast (DIC) microscopy. The ventral neurite was scored if it had any ventral filopodia or if a leading edge formed during the middle L3 stage, and the most prevalent direction of growth was noted. Only one HSN was counted as having a non-ventral neurite per animal. Images were taken using epifluorescent microscopy with a Zeiss 63× water immersion objective.

### Analysis of the axon phenotype in L4 stage animal

For analysis of the AVM axon protrusion phenotype, L4 stage larvae were mounted on a 5% agarose pad with 10 mM levamisole buffer. The AVM axon was visualized in L4 stage with the transgenic strain *zdIs5 [mec-4::GFP]*. Axons were scored as anterior protrusion if the axon extended laterally more than three cell body lengths from the cell body. Axons were scored as having dorsal or posterior protrusion if the axon extended dorsally or posteriorly for a distance greater than two cell body lengths from the cell body. The AVM was considered multipolar if more than one process, greater than one cell body length, was observed. Images were taken using epifluorescent microscopy with a Zeiss 40× objective.

For analysis of the HSN axon protrusion phenotype, L4 stage larvae were mounted on a 5% agarose pad with 10 mM levamisole buffer. HSN axons were visualized using the transgenic strain *kyIs262[unc-86::myr-GFP]*. An anterior protrusion was scored if the axon extended from the anterior side of the cell body for a distance greater than the length of three cell bodies. A dorsal or posterior protrusion was scored if the axon extended dorsally or posteriorly for a distance greater than two cell body lengths. The HSN was considered multipolar if more than one process extended a length greater than one cell body. Images were taken using epifluorescent microscopy with a Zeiss 40× objective.

### Analysis of the UNC-40::GFP localization in L2 stage animal

For analysis of UNC-40::GFP localization, L2 stage larvae with the transgenic marker *kyEx1212[unc-86::unc-40::GFP; odr-1::dsRed]* were mounted on a 5% agarose pad with 10 mM levamisole buffer. Staging was determined by the gonad cell number and gonad size under differential interference contrast (DIC) microscopy. Images were taken using epifluorescent microscopy with a Zeiss 63× water immersion objective. The UNC-40::GFP localization was determined by measuring the average intensity under lines drawn along the dorsal and ventral edges of each HSN cell body by using ImageJ software. For analysis of the anterior–posterior orientation of UNC-40::GFP, the dorsal segment was geometrically divided into three equal lengths (dorsal anterior, dorsal central and dorsal posterior segments). The line-scan intensity plots of each of these segments were recorded. ANOVA (http://www.physics.csbsju.edu/stats/anova.html) test was used to determine if there is a significant difference between intensities of three segments. The dorsal distribution was considered uniform if p≥0.05 and was considered asymmetrical if p≤0.05. Within an asymmetric population, the highest percent intensity was considered to localize UNC-40::GFP to either anterior, posterior or central domain of the dorsal surface.

### Simulations

A program to simulate a two-dimensional lattice random walk based on the probability of dorsal, ventral, anterior, and posterior outgrowth for a mutant (Tables 1 and 2) was created using MATLAB. (The directions of the axons from multipolar neurons were not scored. These axons appear to behave the same as the axons from monopolar neurons, but this has not yet been rigorously tested.) The probability of dorsal, ventral, anterior, or posterior outgrowth was assigned for the direction of each step of a random walk moving up, down, left or right, respectively. Each variable is considered independent and identically distributed. Simulations of 250 equal size steps were plotted.
